# Professional identity, career choices, and working conditions of future and young dentists in Germany – study design and methods of a nationwide comprehensive survey

**DOI:** 10.1186/s12903-017-0417-y

**Published:** 2017-10-18

**Authors:** Nele Kettler, Nicolas Frenzel Baudisch, Wolfgang Micheelis, David Klingenberger, A. Rainer Jordan

**Affiliations:** Institute of German Dentists (IDZ), Universitaetsstrasse 73, 50931 Cologne, Germany

**Keywords:** Professional identity, Career choices, Working conditions, Dental professional’s research, Workforce, Dental students

## Abstract

**Background:**

Little is known regarding young and future dentists’ career choices, professional identity, and working conditions in Germany. While the dental healthcare environment and demands in treatment are changing, it remains unclear what job perceptions young dentists have developed at the beginning of their work life and if and how these perceptions change during the subsequent years. The aim of this study was to survey future and young dentists regarding their professional identity, planned career paths, and working conditions and strains to understand career decisions and choices and enable policy makers to include future dentists‘ views and expectations in their decisions.

**Methods/design:**

This study is a longitudinal nationwide survey over a time span of 4 years of dental students and young dentists in Germany and is comprised of three waves. The first wave focuses on dental students in their final year before the state examination and is composed of a qualitative pre-study in the form of focus groups and a quantitative main survey in the form of a questionnaire. The end points were established to analyse (1) the professional identity of the young future dentists; (2) their career paths, preparation for a career, and basic career conditions; and (3) perceived conditions and strains. The aim of the overall survey was to depict the development of these three aspects during the first years of work life. All of the questions were evaluated with a descriptive univariate analysis. The analysed subgroups were grouped according to gender, target working condition (employed/self-employed), and primary socialisation (parents dentists/parents not dentists).

**Discussion:**

To our knowledge, this is the only study which focuses on career choices, professional identity, and working conditions of future and young dentists in Germany. The longitudinal observation provides information that is essential for professional and purposive dental health care planning, and to meet the oral health demands and needs of the German population appropriately over the long term.

**Trial registration:**

German Health Services Research Data Bank VfD_Y-Dent_14_003759.

## Background

Little is known regarding young and future dentists’ career choices, professional identity, and working conditions in Germany. After completing their final examination, young dentists emerge into a changing dental healthcare environment. These changes are caused by both societal development and governmental intervention. Since the Statutory Health Insurance Doctors‘and Dentists‘Rights Amendment Act (VÄndG) came into effect in Germany in 2007, the employment of dentists for an unlimited time period is now possible. Since then, the number of employed dentists has risen continually from 1986 in 2007 to 9695 in 2015 [1]. Two-thirds of the employed dentists are female [2]. Prospectively, female dentists will outnumber male dentists within the next decade [3], and the professional profile and mode of working will likely be affected by female views [4]. Not only the profession itself but also demands in treatment have changed; for example, the ageing population and achievements in prevention-oriented dentistry have evoked the need for altered delivery of health care in the future [5].

Dental students are prepared for this vocational field during their academic education, and the necessary skills and competencies are learned and trained for at the university. In the winter semester of 2014/2015, 15,020 students were enrolled in dentistry programmes in Germany [2, 6]. The minimum period of study in Germany is 10 semesters, and one additional semester is required to take the state examination. Not only skills and knowledge but also internalisation of professional norms, the competence to make and explain decisions, and the ability to interact appropriately, bear the strains of the position, and control affect are adopted during this time [7]. In this process, the so-called anticipatory socialisation, the standards and values of the dental profession are adopted [8], which leads to a comparatively homogeneous professional group [4, 9]. At the end of dental academic education, the students enter a status of transition into the beginning of the occupational phase, and socialisation *in* the profession follows socialisation *into* the profession [10]. After finishing the state examination, young dentists are usually required to complete 2 years of an approved supervised, salaried assistantship with a registered statutory health insurance dentist. In 2014, 4747 dentists were registered as assistants (excluding the federal state of Schleswig-Holstein) [11]. A second status transition into the next professional phase follows, during which the medium-term future career is usually planned. After their assistantship, dentists are allowed to work as fully trained employees or establish their own practice as a statutory health insurance dentist (establishment of a private practice does not require completion of the assistantship). However, it remains unclear which professional identity young dentists have developed, which career path they have chosen, and how working conditions are perceived at the beginning of their first status transition from dental student to assistant and if, why, and how these perceptions change during the following status transitions.

In other countries, research has been conducted on the career views or job perceptions of dental students, often at selected universities [12–18]; however, no nationwide study on the reasons for studying dentistry, career expectations, and future challenges in this phase of life has been conducted in Germany. Even though similarities in dental students’ views and prospects can be observed between countries, to a certain degree [19, 20], studies on German future dentists’ job perceptions and career plans are essential for well-directed planning of German healthcare needs.

The importance of knowledge regarding the intentions of future workforce members has already been identified for the medical field in Germany, and these selected studies focus on future doctors’ perceptions of their working environment, their specialisation plans, their willingness to work in different and underserved regions, and their demands related to life-domain balance [21–24]. Of the few existing studies in Germany concerning the job profile of dentists or planning of dental career paths, none focuses on students or young dentists, but on the role perceptions, medical culture, and working conditions of employed and self-employed dentists [4] or on female dentists’ gender-specific demands [25, 26].

The findings of these surveys do not provide a full overview of the career goals or values of the next generation of young dentists in Germany. Against the background of a changing vocational field, it is important to gain a comprehensive knowledge on the views of the dental profession, career decisions, perceived conditions, and the prospect of future and young dentists. On the one hand, detailed information might help policy-makers understand dentists’ career decisions, to further the development of realistic views, and to reduce possible concerns or barriers. On the other hand, a deep level of information is essential for dental health care planning to meet the oral health needs of the German population.

In the current longitudinal nationwide study, future and young dentists were surveyed regarding their professional identity, planned career paths, and working conditions and strains to understand career decisions and choices and enable policy makers to include future dentists’ views and expectations in their decisions.

## Methods

### Study design

**Fig. 1:**
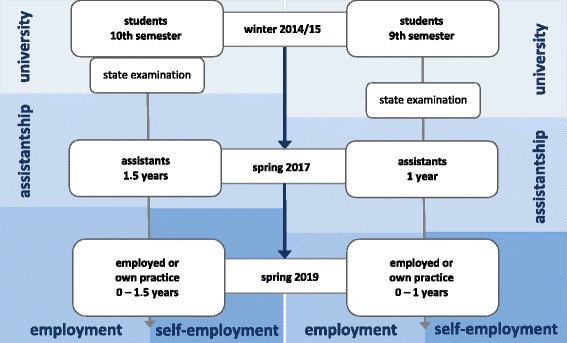
Timetable of the longitudinal study

The study is a longitudinal nationwide survey of dental students and young dentists in Germany and comprises three waves. Participants are accompanied over a time span of 4 years starting at the end of their academic dental studies, continuing during their assistantship and extending until they begin working as an employee or as a self-employed dentist in their own practice (Fig. 1).

The first wave focuses on the dental students in their final year before the state examination and is composed of a qualitative pre-study in the form of focus groups and a quantitative main survey in the form of a questionnaire. In winter semester of 2014/15, all of the dental students attending courses of the 9th and 10th semester at all universities offering dentistry as a subject of study in Germany were invited to fill out a questionnaire via student representative bodies. A total of 69.3% of all contacted dental students participated, and the participation rate varied from 42.2% to 97.5% depending on the university. In the second and third waves, the same students will be surveyed again online, for organisational reasons. The methodological details of the first wave and the longitudinal concept are described below.

### Qualitative study

#### Study design development and purpose

The qualitative study was conducted as a pre-study in the form of focus groups. The results were to (1) determine the most relevant aspects of the general topic, (2) provide evidence of appropriateness and adequacy of ex-ante generated assumptions of the main study, and (3) gain information on the participants’ life work context to facilitate the interpretation of the results of the quantitative survey.

The focus group guideline was developed based on the endpoints of the general study. The general concept of the guideline was developed at the Institute of German Dentists (IDZ) and finalised by an external project partner (Institut fuer Marktforschung im Gesundheitswesen [IMIG], Munich, Germany).

After appraising two focus groups, it became apparent that attitudes and motives regarding career paths between female and male students differed on several points. Therefore, a third focus group was conducted with female students only because they currently represent the majority of dental students in Germany.

### Setting

Focus groups were carried out in summer 2014 at Ludwigs-Maximilian-University Munich, the University of Cologne, and Martin-Luther-University Halle-Wittenberg. The focus groups were assembled and led by the IMIG.

The focus group in Munich took place in July 2014; *n* = 6 female and *n* = 3 male students participated. The focus group in Cologne took place in July 2014; *n* = 4 female and n = 3 male students participated. The third focus group was conducted in Halle/Saale in August 2014 with *n* = 8 female students. The focus group exchanges took approximately 2 h. The contents were audiotaped, and an anonymous verbatim transcript was subsequently written.

### Recruitment

Participants of the 9th and 10th semester were recruited via student representative bodies. The students received reimbursement for their participation. Cologne, Munich, and Halle were chosen as the locations due to their different geographical positions in Germany and their proximity to the institutes performing the study.

### Analysis

The contents of the focus groups were analysed according to Mayring [27]. First, the different aspects of the general topic were determined. An inductive approach was chosen: the transcribed report was analysed exploratively and tenor groups were built in regards to content. The correct attribution of the tenor groups was checked, and the categories were built to represent the central contents of the focus groups. Second, the appropriateness and adequacy of ex-ante generated assumptions were reviewed with a deductive approach: a category system corresponding to the current state of the research and existing theories was developed. By filtering the contents, it was possible to assign text components to equivalent categories.

### Quantitative study

#### Study design development

The quantitative main study was conducted in the form of a paper-and-pencil questionnaire. The results of the qualitative pre-study were utilised during the development of the questionnaire. Furthermore, the Institute of Occupational and Social Medicine of Heinrich-Heine-University Duesseldorf and the `profession, family, and practice management‘ committee of the German Dental Association (BZAEK) were consulted during development of the questionnaire.

The longitudinal study concept aims to survey the same students three times within 4 years. The first wave focuses on the dental students at the end of their academic studies. The second wave surveys the same participants 2 years later in the assistantship. Two years later, the third and final wave will be conducted when the former students are working as employees in statutory health insurance or private practice, have established a practice of their own, or have chosen a different (career) path.

### Study setting

A total of 29 public universities and one private university in Germany offer dentistry as a subject of study. The survey took place at all dental university departments in Germany between the last week of November 2014 and the first week of March 2015. In the winter semester of 2014/2015, a total of 15,020 people [2, 6] were studying dentistry in Germany. Of those, 1972 were enrolled in courses of the 9th or 10th semester. According to the data provided by the student representative bodies, the number of students varied from 21 to 105 depending on the university location. A printed version of the questionnaire was handed out to eligible students at each university via student representative bodies and semester representatives (usually during lectures). Field time ended on March 6th, 2015. All of the questionnaires arriving at IDZ after that point were not included in the analysis.

### Study end points

The end points of the quantitative main study of the first wave were to analyse (1) the professional identity of young future dentists, (2) their career paths, preparation for career, and basic career conditions, and (3) perceived conditions and strains.

The aim of the overall survey was to depict the development of these three aspects during the first years of work life.

### Recruitment and participants

**Fig. 2:**
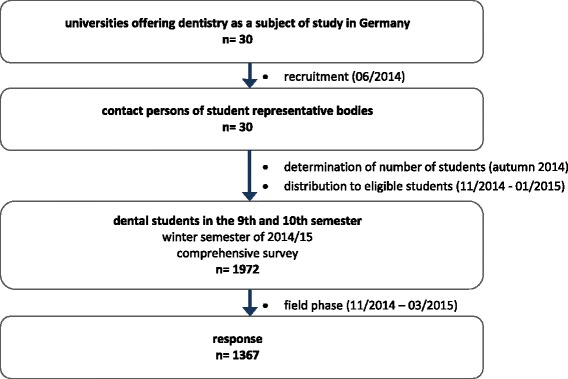
Flowchart of participants

The study was intended as a general survey of all future dentists’ views in its entirety. As a total population survey of all dental students in their final year was feasible and generates more comprehensive data than a sample, the main study was conducted as a comprehensive survey of dental students. Eligible participants were all dental students attending courses of the 9th and 10th semester. No exclusion criteria were defined (e.g., age or nationality). As the survey was intended as a whole-population survey, we did not calculate an intended sample size. A participant flow chart is presented in Fig. 2.

No publicly available general register enlisting all of the students exists, and for reasons of data protection, universities do not disclose personal data of students, e.g., private addresses. It was therefore not possible to organise the study as a postal survey; instead, allocation via student representative bodies was conducted. On a biannual meeting of dental student representative bodies in June 2014 the study design was presented to the attending representatives. Thereafter, each university was asked to appoint a local contact person for the study. A total of 22 of 30 student representative bodies agreed to participate in the study. The eight remaining student representative bodies were subsequently contacted; the study design and aims were explained to them, and all eight agreed to participate.

### Description of material (questionnaire)

Some studies in the medical and dental literature are known to discuss similar topics, and the findings of some of these studies were used to develop the questionnaire. In a few cases, it was possible to adopt proven and validated survey instruments. Furthermore, findings from the focus groups were incorporated in the survey.

Corresponding to the end points of the study, the questionnaire is composed of three subject areas (Table 1).

**Table 1:** Dimensions of the questionnaire

Dimensions					
Professional identity		Career paths		Conditions and strains	
Categories	*External source*	Categories	*External source*	Categories	*External source*
- self-perception	*Härlen and Kultermann, 2000* [28]	- employment/establishment of own practice	*Hartmannbund, 2012* [23]*; Jacob* et al.*, 2015* [22]	- weighting work life/private life	*Abele* et al.*, 2006* [33]*; Abele* et al.*, 2009* [36]*; Abele and Hagmaier, 2011* [34]
- reasons for studying dentistry	*Härlen and Kultermann, 2000* [28]	- requirements	*Hartmannbund 2012* [23]*; Jacob* et al. *2010* [21]	- demand-control	*Karasek, 1979* [37]
- satisfaction with profession	*Jacob* et al.*, 2010* [21]*; BMA, 2009* [29]	- working regions	*Jacob* et al.*, 2015* [22]	- effort-reward imbalance	*Siegrist, 1996* [38]
- challenges	*Bergmann-Krauss* et al. * Die Fortbildung des niedergelassenen Zahnarztes. Nutzen und Bewertung, unpublished. Schneller and Micheelis, 1997* [30]*; Micheelis* et al.*, 2010* [4]*; Oberlander, 2008* [31]	- further training and specialisation	*Micheelis* et al.*, 2010* [4]	- overcommitment	*Schirmer, 2015* [40]
		- personal and career goals	*Abele* et al.*, 2006* [33]*; Abele and Hagmaier, 2011* [34]	- depression	*Gräfe* et al.*, 2004* [42]
		- job expectations	*Abele* et al.*, 2006* [33]*; Abele and Hagmaier, 2011* [34]		
		- knowledge of career paths	*Jacob* et al.*, 2015* [22]		
		- preparation for career	*BMA, 2009* [29]		
		- occupational self-efficacy expectations	*Abele* et al.*, 2000* [35]		

The first dealt with questions regarding the (1) professional identity of future dentists.

To assess *self-perception* as a specific type of dentist, a typology of the forms of “dental professionalism”, e.g., “passionate craftsman” or “specialised health professional” by Härlen and Kultermann from 2001 provided a basis [28]. Findings from the focus groups were used to refine this typology, as more types had been identified and some were a specification of the primary types described by Härlen and Kultermann. *Reasons for studying dentistry* had been described by Härlen and Kultermann as well and were also supplemented by findings from the focus groups for the questionnaire.

*Satisfaction* with the chosen profession was a topic in a few of the previous surveys: a question regarding willingness to study the subject again was adopted from a nationwide survey with medical students [21]. Another question was based on a survey with postgraduate students in England who had been asked about the strength of their desire to practise medicine [29].

*Challenges* of the profession are a recurring subject in dental health profession research. One question in our survey inquired about pleasant and unpleasant issues in dentistry, and a similar question was part of nationwide surveys conducted 10 (Bergmann-Krauss et al: Die Fortbildung des niedergelassenen Zahnarztes. Nutzen und Bewertung, unpublished) and 19 years [30] ago. Challenges might also result from the conditions of the health system, which can restrict autonomy. A corresponding question was modified from a regional [31] and a national survey [4] according to the results of the qualitative pre-study and incorporated in the questionnaire.

The second subject area focused on (2) career paths, preparation for career, and career conditions.

The main career paths in dentistry in Germany include either *employment or establishment of one’s own practice*. For long-term needs and demand-based planning, knowledge on the intended career choices of young dentists is crucial. Therefore, the questionnaire included four questions on this topic based on national surveys with medical students [22, 24] and modified according to the results of the focus groups.

To determine which factors are seen as *requirements* for the decision to establish a practice or work as an employee, prerequisites were discussed in the focus groups. Similar requirements had been observed in medical students [21, 24] and two questions were appropriately modified for the dental students’ questionnaire.

In parts of Germany, a shortage of general medical doctors can be observed [32]. Loosely based on a question in a nationwide survey with medical students [22], we therefore inquired regarding the willingness to live and work in different *regions* and areas of Germany.

Further regular training is required for dentists in Germany, and some take the opportunity to prioritise one or more fields of activity. Questions on *further training* and *specialisation* were adopted and modified from a previous national survey with dentists conducted in 2009 [4].

One question inquired about *personal and career goals* and was influenced by the results of the focus groups and the findings from open questions regarding goals in a long-term cohort study by Abele et al., which accompanied alumni of the University of Erlangen-Nuremberg [33, 34].

The students were asked about their short-term *expectations* regarding the next career-step, i.e., their assistantship. This question was inspired by the results of the group discussions and findings of the longitudinal studies of Abele et al. [33, 34].

Based on a German survey with medical students [22] and the results of the focus groups, one question focused on the *knowledge of career paths*. A question regarding *preparation for career* by the university courses was based on a question in a postgraduate survey of medical students in England [29]. In line with the findings of the focus groups, this question focused less on soft skills, as in the postgraduates’ survey, but rather on the contents of lectures and courses.

In 2000, Abele et al. developed a scale to measure *occupational self-efficacy expectations* to predict success in career start of university graduates [35]. This scale was adopted in the questionnaire.

The third subject area engaged in (3) conditions and strains during dental studies*.* Different approaches exist to measure the extent of strain in work or study environment.

To measure *weighting* between the domains of *work life and private life*, Abele et al. created a scale for their long-term study [33, 34, 36] focussing on private life in general rather than family life [33]. As the focus groups implied, only a few dental students had started a family, yet the question from Abele et al. was adopted in a shortened version for the dental student survey. A perceived weighting of the work life and private life domains was conducted with a graphical illustration [33](p. 36), which was adapted to the dental students’ situation.

One approach to measure the extent of strain is the *demand-control* model. According to Karasek, imbalance between demand resulting from and control of the practised job may result in strain, which may lead to health problems. In this study, demand and control during dental studies was measured with a short version of Karasek’s Job Content Questionnaire [37]. This scale permits the identification of a person who experiences an imbalance between demand and control and therefore shows high strain.

In Siegrist’s *effort-reward-imbalance* model [38], reward or gratification are bestowed during work life, whereas effort can be of either a personal or circumstantial nature. The circumstantial or extrinsic effort refers to job conditions. Effort-reward-imbalance was measured with a scale containing 14 items, which had been tested previously with medical students [39]. The personal or intrinsic effort is called *overcommitment* [40]. Overcommitment is measured with a six-point-scale, which was also applied in this questionnaire.

Imbalance between demand and control or between reward and effort and a high overcommitment cause a higher risk of disease [41]. Disease might manifest as mental disease, for example, disposition to *depression*. A nine-item scale to measure depression was taken from the Personal Health Questionnaire (PHQ), which was found to be a reliable scientific screening tool [42] and can indicate the manifestation of acute psychological disorder.

Furthermore, *sociodemographic data* questions regarding age, gender, and family background were included. Focus group discussions supported the idea that family planning and planning of the professional career are closely related. Therefore, a question on family planning was integrated in the questionnaire based on questions from surveys with medical students [21, 22, 24].

The final questionnaire comprised 34 questions: 6 regarding the professional profile, 14 regarding the career, 6 conditions and strains, and 8 sociodemographics.^1^

The questionnaires are to be applied during the assistantship and 2 years later in employment or self-employment and will be based on this first questionnaire but adapted to the altered work-environment and optimised due to experience of the first wave.

### Longitudinal assignment

To be able to assign the questionnaires longitudinally, but nevertheless pseudonymously, every participating student was asked to generate a personal code. This code consists of five digits: the first two are numbers from the date of birth and the last three letters from the parents’ first names and the place of birth. This distinct, simple, and reproducible combination of letters and numbers guarantees the correct assignment of the questionnaires in the course of the longitudinal study. The generation of the code was explained in the questionnaire, the letter of consent, and the cover letter in detail.

### Pretest

The questionnaire was pretested by dental students of Witten/Herdecke University in October 2014 for coherence, adequacy and feasibility. A total of 17 students attending a 9th semester lecture participated in the pretest. A total of 41 comments on the questionnaire were provided and mainly regarded phrasing and content. Queries disclosed the need for improvement in some of the questions, and on this basis, the questionnaire was subsequently finalised for the field period.

### Data collection methods and management

In October 2014, each local contact person was individually contacted to discuss the study procedure at the universities regarding the number of questionnaires and the methods of allocation. They were asked to provide information on the number of students attending courses of the 9th and 10th semester at their university and an anonymous listing of the ages and genders of these students to generate an overview of the target study population. Packages with envelopes and additional material supporting the study procedure were sent to each university in November 2014. The envelopes were distributed to the students between the last week in November 2014 and the second week in January 2015. At most universities, semester representatives handed out the envelopes in a compulsory lecture, whereas some favoured distribution in seminars or in a particular treatment course.

The questionnaire for the first wave was handed out as a paper version, which was to be sent back to the IDZ via mail. Each student received an envelope containing one white questionnaire, one large, white, post-free envelope addressed to IDZ, one blue letter of consent, and one small, blue, post-free envelope addressed to the National Association of Statutory Health Insurance Dentists (KZBV). Different colours were chosen to simplify correct dispatch of the questionnaire to IDZ (white) and the letter of consent to KZBV (blue).

The letter of consent asked for the students’ permission to be contacted again and for their e-mail address for contacting them after two and 4 years. Information on the e-mail address was optional only for those who wished to be surveyed again and/or receive an incentive. Consent to being surveyed again was not required for receipt of the incentive for participation in the first wave.

The field phase took 6 months, and the students were given several opportunities to return all of the documents: either sending the questionnaire and the letter of consent to IDZ and KZBV, respectively, or by central collection at the universities. In many universities, this option was provided via a locked collection box or the mailbox of the students’ representative body. Some of the questionnaires were handed in to the semester representatives or contact persons, and some of them were collected personally.

The return to two different addresses (IDZ and KZBV) was chosen for data protection: e-mail address and responses in the questionnaire were recorded at different sites. The linkage between the questionnaire and the e-mail address is only possible via the personal code on both documents. The personal code on each returned questionnaire was registered at IDZ. The e-mail address, consent to participate again, and the code were registered at KZBV. The return of the questionnaire was checked via personal code. If the e-mail address had been provided and the questionnaire had been sent back, the appropriate student received an incentive (online voucher).

Student representative bodies received a financial recompense for their assistance when the anonymous listing of the number, age, and gender of the students had been provided, all questionnaires had been handed out, and the method of dispatch of the questionnaires had been settled.

### Quality control

To check for hints of participation biases, the demographic composition of the participants was compared to the demographic composition of the target population. Only a slight deviation in gender (φ = 0.05) and age (φ = 0.1) could be found (Table 2), whereas the percentage of participants of each university varied. Because the survey was designed as an exhaustive sampling and nearly 70% of all of the eligible students participated (42% to 98% of the students at each university), the data were not weighted for analysis.

**Table 2:** Demographic data of the target population and participants

	Target population			Participants
	All^a^	Complete cases^b^		
	*n* = 1972	*n* = 1642	*n* = 1485	*n* = 1367
	%	%	%	%
Gender				
Female	54.7	65.7		68.0
Male	28.5	34.3		32.0
Unknown	16.7	0.0		0.1
Age (years)				
≤ 23	10.4		13.9	15.6
24–25	31.7		42.1	44.2
26–27	11.9		15.8	16.0
28–29	10.0		13.3	12.5
30–31	5.6		7.5	6.5
≥ 32	5.7		7.5	5.0
Unknown	31.5		0.0	0.2

^a^all of the dental students attending courses of the 9th and 10th semester in Germany in the winter semester of 2014/15, including those with missing age and gender information

^b^only dental students attending courses of the 9th and 10th semester in Germany in the winter semester of 2014/15 whose data on age and/or gender were provided by student representative bodies

### Statistical methods

Entry of the collected data was provided by an external company (AFEK Analysen, Forschung, Engineering, Kommunikationstechnik, Aachen, Germany). The statistical analyses were conducted at IDZ with the Statistical Package for the Social Sciences (IBM SPSS Statistics, version 22, IBM Germany, Ehningen).

The analysed subgroups were grouped according to gender (male/female), target working condition (employed/self-employed), and primary socialisation (parents dentists/parents not dentists). In the latter question‚ the responses “one parent” and “both parents” for the question regarding dental background were combined for statistical analysis because we presumed that similar anticipation of ideals and moral concepts of the profession during primary socialisation occurred in both cases.

All of the questions were analysed using descriptive statistics as a first step. The significance of inference statistics was evaluated with Pearson’s Chi-Square test; *p* ≤ 0.05 was set to indicate statistical significance.

The following questions were subjected to further analysis:

1. Professional identity: a factor analysis was conducted with the items for *reasons for studying dentistry*. The method of extraction was principal component analysis, and the method of rotation was varimax with Kaiser’s normalisation. The identified factors were subjected to reliability analysis to evaluate their adequacy.
2. Career: Univariate statistics of the mean, standard deviation, and a 95% confidence interval were calculated for the question regarding *preparation for career*. The Kolmogorov-Smirnov test showed that the variable *preparation for career* was not normally distributed, and differences in the mean for gender and primary socialisation regarding data of *preparation for career* and *life-domain balance* were tested with the Mann-Whitney U-test for significance.
3. Conditions and strains: The variable for *life-domain balance* was analysed in an analogous manner to the variable for *preparation for career*.

The median of both the *demand and control* scale was calculated, and individuals with a demand value above the median and a control value below the median were considered to be in the high-strain condition. Questions regarding *effort-reward imbalance* and *overcommitment* were analysed with a reliability analysis (Cronbach’s α).

Questions regarding *depression* were analysed categorically and dimensionally. For categorical analysis, the pattern of provided answers enables assignment to one of three categories‚ “major depressive syndrome”, “other depressive symptoms”, or “no depressive symptoms”. With dimensional analysis, the severity was measured by adding definite scores for the given answers. The total score ranged from 0 to 27, and the corresponding categories are mild, moderate, moderately severe and severe depression.

## Discussion

To our knowledge, this is the only study to date to focus on the career choices, professional identity, and working conditions of future and young dentists in Germany.

The professional identity of dentists has been subjected to previous studies [4, 28], but never over a period of time covering several status transitions. The longitudinal design offers the opportunity to observe short-term changes in individuals’ own professional perception on a personal level between career stages and to observe long-term changes in the general professional perception by comparing selected results to those of previously conducted studies.

To date, the careers of young dentists in Germany could only be traced by reports of establishment or sale of practices and registers of employed dentists. To what extent these careers correlate to earlier career plans and what factors influence the pursuit or rejection of the plans is unknown. The longitudinal study design can help to understand career paths and decisions of young dentists and reveal possible barriers for establishment of one’s own practice.

Imbalance between work life and private life can lead to dissatisfaction, imbalance between demand and control, and between effort and reward in work life may lead to a higher risk of disease [37, 38]. If and to what extent an imbalance exists during the early years of professional life and how it changes during different professional status will be observed in this study.

One strength of the study is its conceptualisation as a comprehensive survey. Every dental student in Germany attending courses in the 9th and 10th semester was given the opportunity to participate. The findings might be distorted as the response rate of the questionnaire ranged from 42% to 98% depending on the university. Nevertheless, it can be assumed that a nationwide total survey with nearly a 70% overall response rate provides a reliable picture of the views of future dentists in Germany. Another strength is the mixed-methods approach: the dimensions were examined quantitatively and qualitatively, and the perspectives triangulated. Thereby, it was possible to improve the questionnaire in advance, generate findings more comprehensively compared to just using one method, and interpret quantitative results guided by the qualitative results.

Organisation of the study procedure, with the help of the student representative bodies, showed advantages and disadvantages. It cannot be verified that every eligible student actually received a questionnaire. Not all contacted persons were able to provide information on the exact number of students in the 9th and 10th semesters; the participation rate can therefore only be approximately determined. However, most student representative bodies and contact persons were very committed to the project, which resulted in high response rates of over 90% at some universities.

Because the students were first surveyed at the end of their academic studies, we have no knowledge on the development and possible changes of their professional profile or their aims or expectations during their academic socialisation. This lack of knowledge was compensated for by comparing students with one or both parents from the dental profession with students whose parents are not dentists. The former might already have internalised values and ideals of the dental profession in their primary socialisation, whereas the latter presumably made contact with those values and ideals for the first time later during their academic studies. This enabled us to rudimentarily retrace the effects of the anticipatory socialisation during their study period.

While a national comparison is only possible for dentists with more professional experience or cross-professionally to medical students, international comparisons of dental students’ perceptions, career options, and studying conditions can be drawn.

International studies state that for some dental students, the reasons for choosing dentistry as a career include a good life balance, financial security and job security, and the opportunity to work with or help other people [16, 18, 43]. Those reasons for studying dentistry seem to be closely related to the developed job profile in later work life [9, 28]. Though a certain group of discontented dentists or (dental) students is identified in different studies [4, 16, 24, 28], most students surveyed in the past seemed to be satisfied with their choice of career [13, 15, 16, 21]. While a significant proportion of medical students in Germany consider career paths outside of healthcare [22, 24], most UK dental students intend to work in a general practice [13, 15]. In international studies, the number of dental students intending to specialise or wanting to work as a dentist with a special interest is higher than those intending to work as a general dental practitioner [12, 14–17]. While Indian dental students are satisfied with their teachers [16], students in the UK experience problems with difficult teaching staff alongside other problems, such as financial worries or lack of confidence [13].

The national and international comparison of expectations, desired career paths, and conditions might help provide a more accurate interpretation of the results of the present study and indicate which conclusions and challenges arise for the dental profession.

Young dentists go through different status transitions in their early job life, usually from academic studies to assistantships to employment or self-employment. The longitudinal study design allows an insight into this presumably most formative professional phase, in which various factors have an influence on development of professional opinions within only a few years: the course for the professional career is set, attitudes and opinions are focused, and influencers within the profession (professors, first employers and new colleagues) affect these opinions. Attitudes and opinions and their development during this time can be assessed, as can possible conflicts of aims or strains. Knowledge of self-perception of young dentists might help to understand career decisions and barriers, perceived options, and demands.

This study may help to enable policy-makers to find approaches to young dentists in the appropriate setting. It will make it possible to respond to their demands directly if necessary and justified to improve conditions and to reduce concerns and barriers. Deficiency in knowledge, once obvious, can be antagonised selectively, and a realistic view of the profession and its professional requirements can be provided to reduce barriers and potential disappointment throughout the career path.

This longitudinal observation provides information on self-perception, career plans, and perceived conditions of young dentists and their development, which is essential for professional and purposive dental health care planning and to meet oral health demands and the needs of the German population appropriately over the long term.

## Footnotes

1 Please contact the corresponding author for a copy of the questionnaire (in German)
